# Effects of Ashwagandha (*Withania somnifera*) on Physical Performance: Systematic Review and Bayesian Meta-Analysis

**DOI:** 10.3390/jfmk6010020

**Published:** 2021-02-11

**Authors:** Diego A. Bonilla, Yurany Moreno, Camila Gho, Jorge L. Petro, Adrián Odriozola-Martínez, Richard B. Kreider

**Affiliations:** 1 Research Division, Dynamical Business & Science Society—DBSS International SAS, Bogotá 110861, Colombia; luzyuranymoreno@gmail.com (Y.M.); camilagho@dbss.pro (C.G.); jlpetro@dbss.pro (J.L.P.); 2 Research Group in Biochemistry and Molecular Biology, Universidad Distrital Francisco José de Caldas, Bogotá 110311, Colombia; 3 Research Group in Physical Activity, Sports and Health Sciences (GICAFS), Universidad de Córdoba, Montería 230002, Colombia; 4 kDNA Genomics, Joxe Mari Korta Research Center, University of the Basque Country UPV/EHU, 20018 Donostia, San Sebastián, Spain; adrianodriozola@gmail.com; 5 Sport Genomics Research Group, Department of Genetics, Physical Anthropology and Animal Physiology, Faculty of Science and Technology, University of the Basque Country (UPV/EHU), 48940 Leioa, Spain; 6 Phymo Lab, Physiology and Molecular Laboratory, 08028 Barcelona, Spain; 7 Exercise & Sport Nutrition Laboratory, Human Clinical Research Facility, Texas A&M University, College Station, TX 77843, USA; rbkreider@tamu.edu

**Keywords:** herbal supplements, muscle strength, cardiorespiratory fitness, exercise tolerance, quality of life, sleep latency

## Abstract

Ashwagandha (*Withania somnifera*) is considered a potent adaptogen and anti-stress agent that could have some potential to improve physical performance. This preferred reporting items for systematic reviews and meta-analyses (PRISMA)-based comprehensive systematic review and Bayesian meta-analysis aimed to evaluate clinical trials up to 2020 from PubMed, ScienceDirect, and Google Scholar databases regarding the effect of Ashwagandha supplementation on physical performance in healthy individuals. Besides implementing estimation statistics analysis, we developed Bayesian hierarchical models for a pre-specified subgroup meta-analysis on strength/power, cardiorespiratory fitness and fatigue/recovery variables. A total of 13 studies met the requirements of this systematic review, although only 12 were included in the quantitative analysis. A low-to-moderate overall risk of bias of the trials included in this study was detected. All Bayesian hierarchical models converged to a target distribution (Ȓ = 1) for both meta-analytic effect size (μ) and between-study standard deviation (τ). The meta-analytic approaches of the included studies revealed that Ashwagandha supplementation was more efficacious than placebo for improving variables related to physical performance in healthy men and female. In fact, the Bayesian models showed that future interventions might be at least in some way beneficial on the analyzed outcomes considering the 95% credible intervals for the meta-analytic effect size. Several practical applications and future directions are discussed, although more comparable studies are needed in exercise training, and athletic populations are needed to derive a more stable estimate of the true underlying effect.

## 1. Introduction

Traditional botanical knowledge is characteristic of cultural contexts with long experience in the natural environment; however, as it is transmitted from generation to generation, it adapts and transforms through scientific experimentation. Within the traditional medicine of India (Ayurveda), the Rasavātam provides nutritional and behavioral recommendations for all phases of life, which may delay the onset of aging processes [1]. Thus, there is a wide range of Rasavātam preventive or therapeutic strategies with various health objectives (e.g., improvement of the libido). One of the most popular plants in Ayurveda is Ashwagandha (*Withania somnifera*), which is considered a potent adaptogen and anti-stress agent [2]. Brekhman and Dardymov [3] proposed in 1969 the term adaptogen as an innocuous agent that increases in a nonspecific way the resistance against harmful factors or physical, chemical, biological and psychological “stressors”, normalizing the homeostasis of individuals. Ashwagandha, commonly known as Indian ginseng or poison gooseberry, belongs to the Solanaceae family (NCBI:txid126910). In addition to its adaptogenic properties, several authors have attributed multiple medicinal benefits including antitumor, anti-inflammatory, hypoglycemic and antioxidant effects [4,5]. These characteristics have generated a great scientific interest in the study of the chemical composition of this plant. Several bioactive compounds, including flavonoids, tannins, alkaloids, glycosides and steroid lactones have been identified in leaves, stems and roots [6]. Within the steroid lactones are the withanolides, which have been described as the main secondary metabolites responsible for the beneficial properties of this plant.

Withanolides are polyoxygenated steroids consisting of a 28C ergostane backbone. The structural diversity of withanolides generally depends on the nature and number of oxygenated substitutes and the degree of saturation of the rings. These compounds can be classified into two main groups based on the C17 side chain, those containing an *δ*-lactone or *δ*-lactol between C22 and C26, and those with an γ-lactone that usually forms between C23 and C26. The first group can be further classified into withanolides with intact (withaferin A) and modified (fisalin C) backbone. Currently, the presence of withanolides with unmodified skeletons has been commonly identified in nature, of which approximately 580 have been reported in the Solanaceae family. The wide structural variety of withanolides may be related to the multiple biological functions that have been described for Ashwagandha: antimicrobial, anti-inflammatory, immunomodulatory, neuroprotective, cytotoxic, and antioxidant, among others [5]. However, several experimental strategies have been developed for the extraction and purification of these compounds. Recently, Nile et al. (2019) compared different methods of withanolide and withanoside extraction from Ashwagandha leaf and root extracts and the effects on their biological activity. The authors demonstrated that subcritical water extraction allowed higher yield in solubilization of these compounds mainly in root extracts, maintaining their biological properties, compared to other methods such as maceration, Soxhlet extraction, and microwave-assisted extraction, which require long periods of time and the use of organic solvents [7]. Trivedi et al. (2017) identified a total of 43 withanolides in the hydroalchoholic extract of Ashwagandha root using LC/MS, GC/MS and NMR, including withaferin A, withanolide A, withanoside IV and VI, withanolide D, dihydrowithanolide D and withanolide sulfoxide [8]. Of these, withaferin A and withanolide A possess therapeutic properties for the treatment of cancer and neurodegenerative diseases, such as Parkinson’s and Alzheimer’s [9,10,11].

Although it has been used therapeutically for a number of reasons, Ashwagandha supplementation lacks sufficient information to be considered effective for all proposed conditions [4]. However, several recent systematic reviews and meta-analyses have shown its potential and safety for controlling anxiety [12], fighting male infertility [13], improving the function of the reproductive system [14], serving as an adjuvant to the treatment of diabetes [15] and avoiding the deterioration of cognitive function [16]. However, it seems that some of the secondary metabolites of Ashwagandha could have some potential at the level of physical performance improvement, being responsible for various effects at the metabolic and physiological level through the regulation of certain anti-inflammatory and anti-oxidative pathways [4,17]. Considering that nutrition is one of the fundamental pillars to optimize sports performance, some have suggested that Ashwagandha may provide ergogenic benefits for active individuals and athletes. In fact, Pérez-Gómez et al. [18] recently reported that consumption of this herbal extract significantly increases maximum oxygen uptake (VO_2max_), although this meta-analysis has some limitations that warrants caution when interpreting results. On the other hand, some previous work has shown that the administration of Ashwagandha during a program of resistance training improves the strength and muscular power of the upper and lower limbs [19,20].

Despite the above, no meta-analytic study has been conducted to evaluate the effect of Ashwagandha supplementation on physical performance. Moreover, no comprehensive systematic review with Bayesian meta-analysis has been carried out on clinical trials that have investigated the effect of the aqueous extract of the root of this plant on the different physical capacities and variables related to physical performance in healthy adult subjects. Thus, this systematic review and meta-analysis evaluated clinical trials up to 2020 that have assessed the effect of Ashwagandha (*Withania somnifera*) supplementation on physical performance (considering as main variables the muscle strength, VO_2max_, muscle fatigue, tiredness, and physical recovery (i.e., pain, inflammatory status, and sleep quality)) in healthy individuals.

## 2. Methods

### 2.1. Protocol and Registration

The present systematic review was conducted and reported according to the established guidelines of the preferred reporting items for systematic reviews and meta-analyses (PRISMA) guidelines [21]. As this review was not eligible to be registered on PROSPERO, the summary information was uploaded to *Figshare* to make it publicly accessible in order to avoid unnecessary duplication (doi:10.6084/m9.figshare.12725081).

### 2.2. Eligibility Criteria

The inclusion criteria for this systematic review were as follows: (1) clinical trials (randomized or not) in healthy females and males; (2) articles that were published from 2010 onwards; (3) studies written in English, German and Spanish; (4) trials that assessed the effect of Ashwagandha supplementation compared to a control group or with repeated measures; and (5) trials that reported the effects on variables related to physical performance (e.g., muscle strength, VO_2max_, muscle fatigue, tiredness, and physical recovery). Studies that did not correspond to original research (e.g., editorials, notes, reviews, dissertations, etc.) or include adults (e.g., children, elderly, etc.) were excluded.

### 2.3. Information Sources

The following academic and free research databases were selected to examine the literature: PubMed/Medline, ScienceDirect, and Google Scholar. Further papers were sought by hand-searching.

### 2.4. Search Methods

The following Boolean algorithms were used to perform the search: PubMed/MEDLINE (ashwagandha OR withania somnifera OR indian ginseng OR winter cherry OR poison gooseberry) AND (performance OR sports OR strength OR recovery OR sleep) NOT a disease; and ScienceDirect (ashwagandha OR withania somnifera) AND (performance OR sports OR strength OR recovery). The identification of potential studies was enriched by performing a hand-search in Google Scholar with free language terms related to Ashwagandha supplementation and physical performance.

### 2.5. Study Selection

After the search of published articles, the filter options of the databases were used to meet the inclusion criteria 1 to 3. After this searching process, the remaining references were filtered by screening the title, abstract, or full-text publication. A network graph was built using the Connected Papers tool (www.connectedpapers.com accessed on 23 June 2020) to ensure the inclusion of recent publications and to visually discover relevant studies. The study selection took place during May and June of 2020, although an updated search was conducted in December 2020 prior to manuscript submission.

### 2.6. Data Collection Process and Items

The full-text articles of the selected studies were evaluated for meeting the inclusion criteria. The following data were obtained and analyzed from the selected studies: (i) descriptive statistics of the study population; (ii) study length; (iii) characteristics of the Ashwagandha supplementation protocol; (iv) magnitude and units of the analyzed variables; (v) the percentage of change according to the formula: ((postpre)/pre) × 100, and significant difference in comparison to placebo or control group (if existed); and (vi) study conclusions.

### 2.7. Risk of Bias

Two authors (D.A.B. and C.G.) independently evaluated the risk of bias of all included clinical trials using the Cochrane risk of bias tool RoB 2.0 [22], including bias due to randomization, bias due to deviations from intended intervention, bias due to missing data, bias due to outcome measurement, and bias due to selection of reported results. All randomized participants in the analysis were included, as it was the least-biased way to analyze clinical effects. Discrepancies were identified and resolved through discussion (with a third author where necessary). The figures summarizing the results of the risk of bias assessment were developed using the risk-of-bias VISualization package (*robvis*) [23].

### 2.8. Summary Measures

The primary outcome was changes in physical performance variables (muscle strength, VO_2max_, muscle fatigue, tiredness, and physical recovery) after Ashwagandha supplementation. Selected publications that met all the requirements went on to the next phase of data analysis and synthesis, where a table of their results and findings comparison was developed and complemented by the review authors considering the items mentioned before (see data items).

### 2.9. Data Analysis and Synthesis

All studies included in this meta-analysis compared an experimental group (i.e., a group that received Ashwagandha supplementation) against a control group (i.e., a group of participants that received a placebo) in regards to physical performance variables. A pre-specified subgroup analysis (strength/power, cardiorespiratory fitness and fatigue/recovery) was conducted to evaluate interaction and effect modification.

In detail, we extracted the changes from baseline (mean change and SDs of the changes) in the experimental interventions (Ashwagandha) and the comparator interventions (placebo). Following the Cochrane Handbook for Systematic Review of Interventions [24], the correlation coefficients (Corr) from studies reported in considerable detail were calculated for each variable in order to impute a change-from-baseline SD in studies for which these values were missing, according to the expression SD change = √(SD^2^_baseline_ + SD^2^_final_ − (2 x Corr × SD_baseline_ × SD_final_)) [25]. We also had e-mail communication with some authors to obtain missing information. Subsequently, the effect sizes, as standardized mean differences, were calculated as unbiased Cohen’s *d* (d_unb_), also known as Hedges’ *g* [26]. Positive scores of standardized mean differences indicated a higher physical performance in each subgroup; however, it should be noted that this method did not correct for differences in the direction of the scale, so to ensure that all the scales point in the same direction, we multiplied some variables by −1 (mainly fatigue/recovery variables), before standardization [24]. There were some complex data structures within the analyzed studies that required attention before computing a summary effect across studies. All solutions to these potential issues were based on the recommendations by Borenstein et al. [27]. On one hand, several studies reported multiple outcomes from the same participants, which may be problematic due to the fact that treating each outcome as a separate unit in the analysis will assign more weight to studies with two outcomes than those reporting one, which leads to a wrong estimate of the precision of the summary effect. To address this problem, we computed the mean of the outcomes for correlated variables in each study and used this synthetic score as the unit of analyses per study. This was different for non-correlated variables given that setting the correlation among the outcomes at 0.00 will treat the comparison as independent of each other and yields the same precision as a separate unit in the meta-analysis. On the other hand, some studies used a single placebo group and several treatment groups which represent certain problems when we want to incorporate both treatment groups in the same analysis. To combine these multiple comparisons within the studies, we set the correlation among comparisons at 0.50.

Finally, to compute effects in each pre-specified subgroup, the meta-analysis was based on d_unb_ between two independent group change-from-baseline means by employing a single pooled standard deviation. The heterogeneity was evaluated by means of the diamond ratio with values approaching or exceeding 2.0, implying considerable heterogeneity. To address any substantial heterogeneity, we checked outlier effects and explored the impact of excluding studies with a standardized mean difference higher than 5 (see Appendix A), considering such big effect sizes highly influence the summary effect. Taking into account the assumption of exchangeability, a random-effect model was used. This first meta-analytic approach was performed within the Exploratory Software for Confidence Intervals (ESCI) [28].

Additionally, to directly model the uncertainty (which has been found to be superior in estimating the between-study heterogeneity and pooled effect) and to produce full posterior distributions (which allows calculating actual probabilities), a Bayesian hierarchical model was used to pool effects in a Bayesian meta-analysis using “Stan” (*brms*) package v2.13.0 [29], within the R statistical computing environment v4.0.2 [30]. Importantly, the effect sizes used in the Bayesian meta-analysis (and displayed in the forest plot) do not correspond to the calculated effect sizes in the original studies (d_unb_), but the estimate of the “true” effect size (*θ_k_*) of each study (obtained from the model with the mentioned function (*brms*). The convergence assessment, that is, if the Hamiltonian Markov chain Monte Carlo algorithm found an optimal solution, was conducted based on posterior predictive checks and with the inspection of potential scale reduction factor (Ȓ) values of the parameter estimates (meta-analytic effect size [μ] and tau [τ]). The posterior distributions of the *θ_k_* for each study, the posterior mean, and the 95% credible intervals (95% CrI) were reported. This last is an interval in which there is a 95% certainty that the “true” value of the parameter lies. The τ value and its squared number (τ^2^) were calculated to report the estimated standard deviation and variance of underlying effects across studies with this meta-analytic approach, respectively. Based on the obtained Bayesian model, we calculated the exact probabilities that μ will be smaller/larger than a given effect size value by looking at the empirical cumulative distribution function (ECDF) of the posterior distribution for the pooled effect size. The function *forest()*, which is included in the *brmstools* package, and the ggplot2 functions were used to draw forest and density plots within R.

## 3. Results

### 3.1. Study Selection

**Figure 1:**
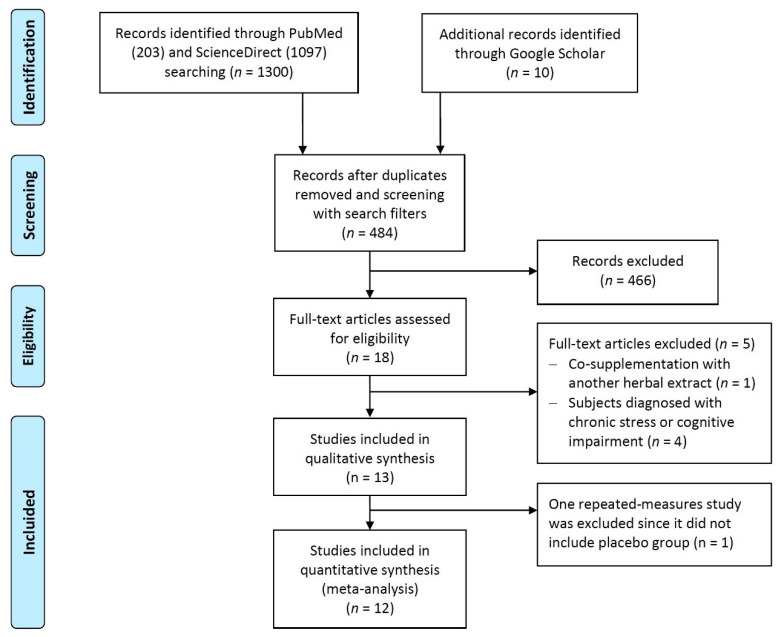
Preferred reporting items for systematic reviews and meta-analyses (PRISMA) flow diagram.

After running the search algorithms with Boolean operators and free language terms, 1310 references were obtained. After the screening process of the publications found (filtering by date, type of article and availability of full text) resulted in 484 potentially eligible studies. However, after evaluating the abstracts and full text of these studies to filter out duplicates and analyze strict compliance with the other inclusion criteria, 471 articles were excluded. A total of 13 studies met the requirements of this systematic review. Figure 1 shows a flowchart of the literature search.

**Figure 2:**
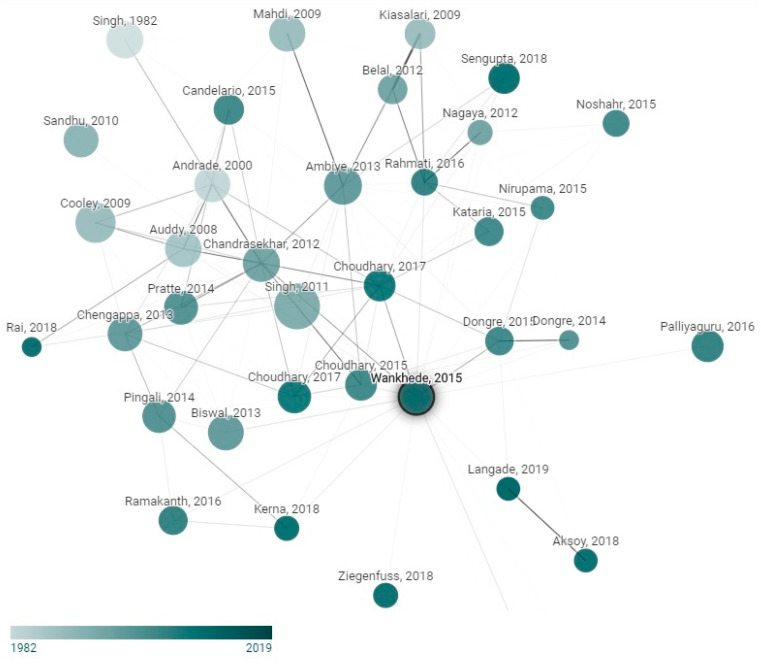
Network graph of the studies related to Ashwagandha supplementation. Node size is proportional to the number of citations, and the color is the publishing year. This graph was developed within www.connectedpapers.com accessed on 23 June 2020.

The verification of relevant publications in the field of supplementation with Ashwagandha, and a visualization of the trends, popular works and dynamics of the field, is shown in Figure 2 through a graph that shows each node as an academic product related to a source document, which was selected according to the number of citations and the high degree of relationship with the objective of this systematic review. Considering the above, the work selected was that of Wankhede et al., 2015 [12].

### 3.2. Risk of Bias within Studies

**Figure 3:**
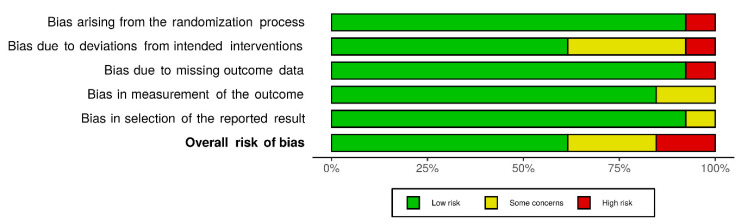
Risk of bias summary for included studies. Weighted bar-chart of the distribution of risk-of-bias judgments. These graphics were obtained using the *robvis* package within the R statistical computing environment.

The methodological quality of the trials included in this systematic review and meta-analysis is presented in Figure 3.

### 3.3. Results of Individual Studies

With a total of 615 healthy adults participating in the studies analyzed in this systematic review, there is direct evidence suggesting that Ashwagandha supplementation has a positive effect on different variables related to physical performance when compared to a placebo or control group. The most relevant aspects of the selected publications are summarized in Table 1.

### 3.4. Synthesis of Results

All studies included in the quantitative analysis were randomized placebo-controlled trials; hence, only one repeated-measures study was excluded since it did not include the placebo group [32]. In addition, both meta-analytic approaches were based on changes from baseline, which according to current literature, might be more efficient and powerful than a comparison of post-intervention values solely, as it removes the between-person variability [24]. Very extreme effect sizes were excluded from the meta-analysis since they may affect the overall effect and/or increase heterogeneity; notwithstanding, we reported all results (including outliers) in Appendix A to demonstrate how the overall effect would have been. In this sense, after excluding outliers, all Bayesian models resulted in Ȓ values equal to one for both parameters (μ and τ), which signified convergence. Additionally, all our Bayesian explorations to quantify posterior distributions with the Hamiltonian Monte Carlo algorithm were performed by running four Markov chains, which implemented 1000 warmup iterations and a total of 4000 post warmup sampling iterations. The following subsections show the pre-specified subgroup analysis.

#### 3.4.1. Strength and Power

**Figure 4:**
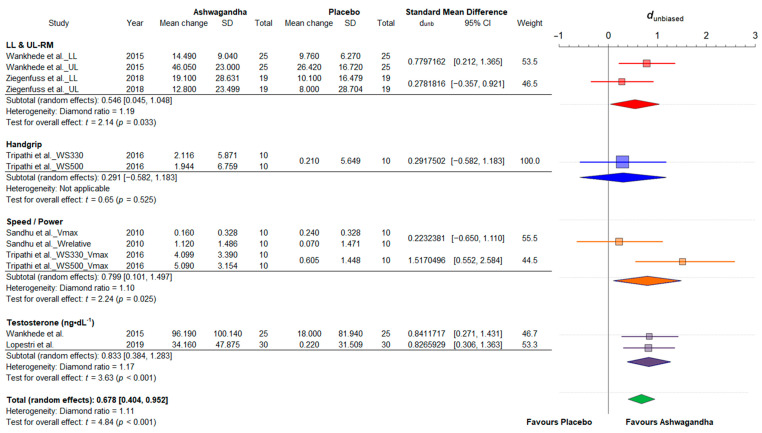
Forest plot depicting the standardized mean differences from the meta-analysis of studies comparing groups with Ashwagandha or placebo on strength/power. Colored squares represent the d_unb_ for individual trials, while colored and green polygons depict the meta-analytic d_unb_ for the indicated subgroups and for the overall (total) results, respectively. Horizontal lines represent the 95% confidence intervals for the data. LL: lower limbs; RM: one-repetition maximum (kg); UL: upper limbs; Vmax: maximum velocity (m·s^−1^); Wrelative: relative power (W·kg^−1^); WS330: *Withania somnifera* 330 mg; WS500: *Withania somnifera* 500 mg.

The quantitative selection of this sub-group meta-analysis included five trials. Three articles [19,20,31] provided multiple outcomes, while one [38] reported both multiple outcomes and multiple treatment arms. In total, seven effect sizes were estimated from different strength- and power-related variables (muscle strength of upper and lower limbs, handgrip, maximum velocity, relative power and testosterone concentration) using data from 198 participants, of which 104 were in Ashwagandha groups and 94 in placebo groups. The results of the random-effects meta-analysis of standardized mean differences for strength/power variables is shown in Figure 4. Compared to placebo, the pooled treatment effect of Ashwagandha supplementation was medium (d_unb_: 0.68; 95% CI: 0.40 to 0.95). There was little heterogeneity among the studies (diamond ratio = 1.11).

**Figure 5:**
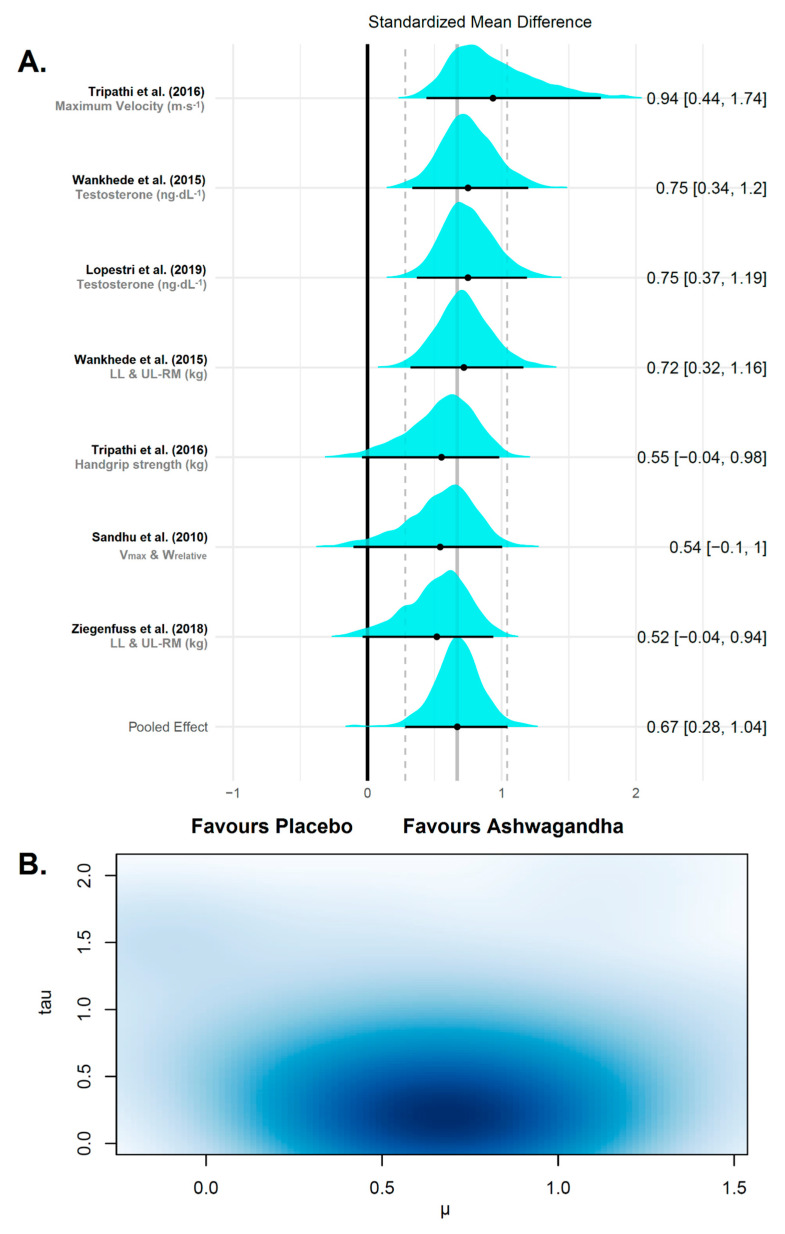
Forest plot of a random effect meta-analysis on strength/power variables after Ashwagandha supplementation. (**A**) The plot shows the names of the studies with their respective variable or multiple comparison/multiple outcomes on the left. On the right are the *θ_k_* and 95% credible interval (CrI). The value of the overall weighted mean of effect sizes is drawn as a vertical line in the middle of the plot, which represents the reference line to test the null hypothesis in each study. The posterior distributions of the estimated effect sizes for each study are shown as cyan densities. The black circle represents the posterior mean, and the horizontal line extending from the point is the 95% CrI. The bottom row is the meta-analytic effect size (µ). (**B**) Multivariate kernel density estimation plot of the posterior distribution of μ (x-axis) and τ (y-axis) with the darker zone indicating increased plausibility of values.

Our Bayesian model (at convergence, Ȓ = 1) revealed an estimate of the pooled effect size of μ = 0.67 with the 95% CrI ranging from 0.28 to 1.04. Therefore, there was a medium overall effect size of the Ashwagandha interventions studied in this subgroup meta-analysis. Relatively small between-study standard deviation was estimated (τ = 0.31 (95% CrI 0.01–0.96)) with an absolute value of the true variance of τ^2^ = 0.09 (low heterogeneity). The ECDF showed that the probability of the pooled effect being greater than 0.20 is very high (98.3%). Therefore, the effects of the Ashwagandha supplementation are very likely to be meaningful on strength/power-related variables. The results of the Bayesian random-effects meta-analysis conducted on the evaluated studies are shown in Figure 5.

#### 3.4.2. Cardiorespiratory Fitness

**Figure 6:**
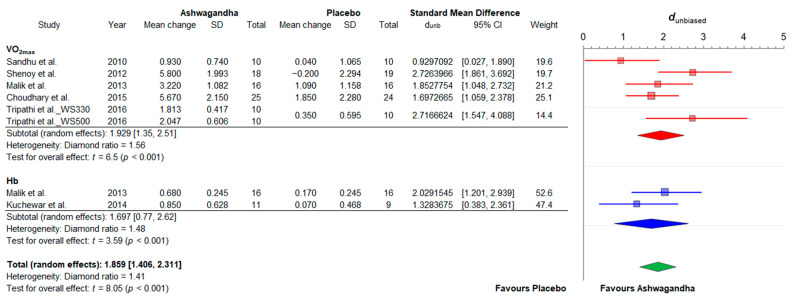
Forest plot depicting the standardized mean differences from the meta-analysis of studies comparing groups with Ashwagandha or placebo on cardiorespiratory fitness. Colored squares represent the d_unb_ for individual trials, while colored and green polygons depict the meta-analytic d_unb_ for the indicated subgroups and for the overall (total) results, respectively. Horizontal lines represent the 95% confidence intervals for the data. Hb: blood hemoglobin concentration; VO_2max_: maximum oxygen uptake; WS330: *Withania somnifera* 330 mg; WS500: *Withania somnifera* 500 mg.

**Figure A1:**
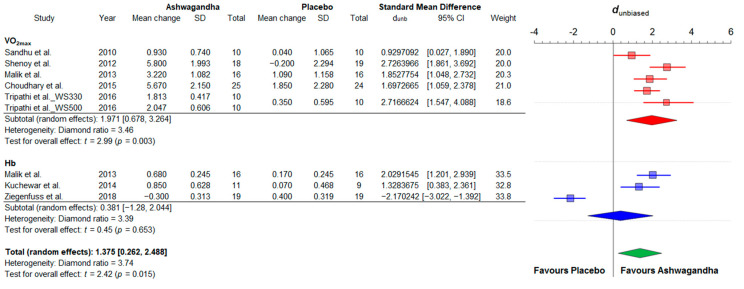
Forest plot depicting the standardized mean differences from a meta-analysis of all studies (including outliers) comparing groups with Ashwagandha or placebo on cardiorespiratory fitness. Colored squares represent the d_unb_ for individual trials, while colored and green polygons depict the meta-analytic d_unb_ for the indicated subgroups and for the overall (total) results, respectively. Horizontal lines represent the 95% confidence intervals for the data. Hb: blood hemoglobin concentration; VO_2max_: maximum oxygen uptake; WS330: *Withania somnifera* 330 mg; WS500: *Withania somnifera* 500 mg.

This sub-group meta-analysis included seven studies. One article [34] provided multiple outcomes, while one study [38] reported on multiple comparisons. A total of eight effect sizes were estimated from research designs that compared Ashwagandha administration (total *n* = 119) with placebo groups (total *n* = 107) on cardiorespiratory fitness-related variables (VO_2max_ and [Hb]). However, this first pooled treatment effect (d_unb_: 1.37; 95% CI: 0.26 to 2.48) was considered as merely exploratory since there was a very high degree of heterogeneity (diamond ratio = 3.74; see Figure A1). Thus, we proceed to the identification of potential outliers and found that the upper bound of the 95% CI of one study [20] was lower than the lower bound o0f the pooled effect CI (extremely small effect size). After exploring the impact of excluding that study, the new heterogeneity was considerably reduced to a medium-to-high-level (diamond ratio = 1.41). The results of this second random-effects meta-analysis of standardized mean differences for VO_2max_ and [Hb] is shown in Figure 6. Compared to placebo, the pooled treatment effect of Ashwagandha supplementation on cardiorespiratory fitness was very large (d_unb_: 1.85; 95% CI: 1.40 to 2.31).

**Figure 7:**
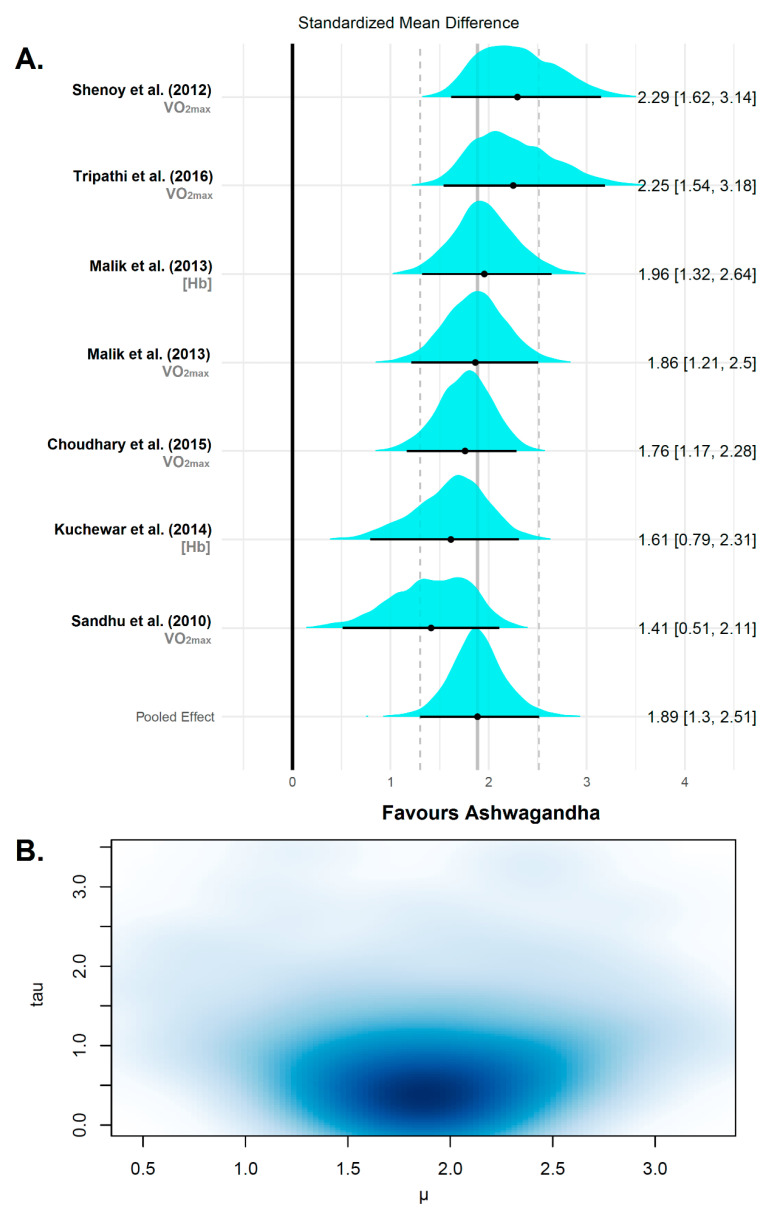
Forest plot of a random effect meta-analysis on cardiorespiratory fitness variables after Ashwagandha supplementation. (**A**) The plot shows the names of the studies with their respective variable or multiple comparison/multiple outcomes on the left. On the right are the *θ_k_* and 95% CrI. The value of the overall weighted mean of effect sizes is drawn as a vertical line in the middle of the plot, which represents the reference line to test the null hypothesis in each study. The posterior distributions of the estimated effect sizes for each study are shown as cyan densities. The black circle represents the posterior mean, and the horizontal line extending from the point is the 95% CrI. The bottom row is the meta-analytic effect size (µ). (**B**) multivariate kernel density estimation plot of the posterior distribution of μ (x-axis) and τ (y-axis) with the darker zone indicating increased plausibility of values.

After confirming convergence (Ȓ = 1), the Bayesian model indicated a very large, pooled effect size that favored the Ashwagandha supplementation (μ = 1.89 (95% CrI − 1.30 to 2.51)). There was a low-to-medium study heterogeneity in terms of between-study standard deviation (τ = 0.55 (95% CrI 0.03–1.44)). However, the inclusion of the outlier effect size within the Bayesian model reduced the pooled effect size (μ = 1.38 (95% CrI − 0.03 to 2.81)) and increased the heterogeneity substantially (τ = 1.91 (95% CrI − 1.03 to 3.54)). The posterior predictive distribution for a future observation showed that the probability of the effect size is greater than 0.20 is absolutely high (100%), which suggests positive effects of Ashwagandha supplementation on cardiorespiratory fitness (i.e., VO_2max_ and [Hb]). The results of the Bayesian random-effects meta-analysis conducted on the evaluated studies of this subgroup are shown in Figure 7.

#### 3.4.3. Fatigue and Recovery

**Figure 8:**
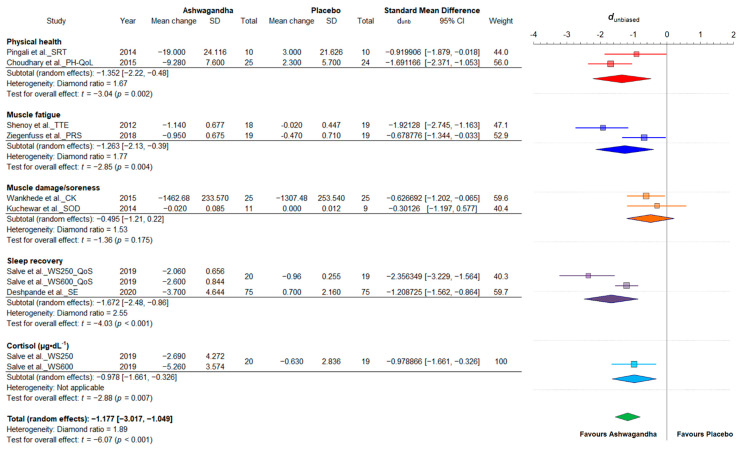
Forest plot depicting the standardized mean differences from the meta-analysis of studies comparing groups with Ashwagandha or placebo on fatigue/recovery. Colored squares represent the d_unb_ for individual trials, while colored and green polygons depict the meta-analytic d_unb_ for the indicated subgroups and for the overall (total) results, respectively. Horizontal lines represent the 95% confidence intervals for the data. SRT: simple reaction time; PH-QoL: physical health component of the quality of life test; TTE: time to exhaustion; PRS: perceived recovery scale; CK: creatine kinase; SOD: superoxide dismutase; SE: sleep efficiency; QoS: quality of sleep; WS250: *Withania somnifera* 250 mg; WS600: *Withania somnifera* 600 mg.

**Figure A2:**
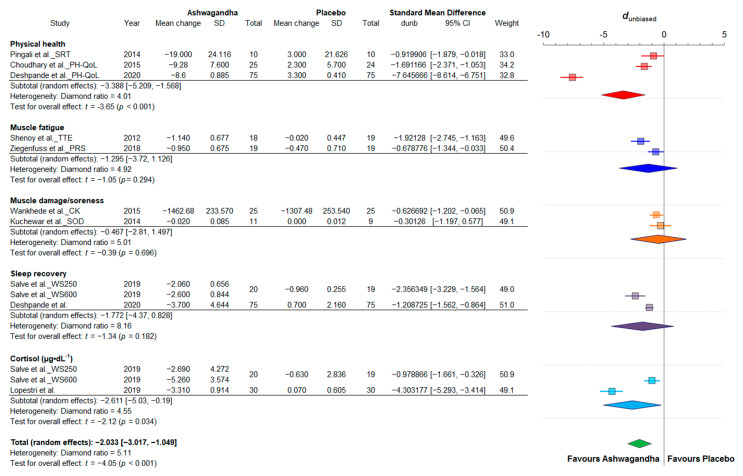
Forest plot depicting the standardized mean differences from a meta-analysis of all studies (including outliers) comparing groups with Ashwagandha or placebo on fatigue/recovery. Colored squares represent the d_unb_ for individual trials, while colored and green polygons depict the meta-analytic d_unb_ for the indicated subgroups and for the overall (total) results, respectively. Horizontal lines represent the 95% confidence intervals for the data. SRT: simple reaction time; PH-QoL: physical health component of the quality of life test; TTE: time to exhaustion; PRS: perceived recovery scale; CK: creatine kinase; SOD: superoxide dismutase; SE: sleep efficiency; QoS: quality of sleep; WS250: *Withania somnifera* 250 mg; WS600: *Withania somnifera* 600 mg.

Taking into account the presence of extremely small effects (the upper limit of the 95% CI was lower than the lower bound of the pooled effect CI), two outliers were identified and were removed from the analysis, leaving a total of nine effect sizes from eight studies. Salve et al. (2019) reported both multiple outcomes and multiple treatment arms [39]. The analyzed outcomes encompassed variables of physical health, muscle fatigue, muscle damage/soreness, sleep recovery, and cortisol from 403 participants, of which 203 were in Ashwagandha groups and 200 in placebo groups. To ensure that all the scales pointed in the same direction in this subgroup meta-analysis of fatigue/recovery-related outcomes, we multiplied several mean differences by −1 (i.e., physical health component of the quality of life test, time to exhaustion, perceived recovery status scale, superoxide dismutase and sleep efficiency); therefore, negative values can be interpreted as counteracting fatigue and favored physical recovery effect. In comparison to the placebo, the pooled treatment effect of Ashwagandha supplementation was very large (d_unb_: −1.17; 95% CI: −3.01 to −1.049) with a medium-high heterogeneity among the studies after removal of the outliers (diamond ratio = 1.89). The results of this random-effects meta-analysis of standardized mean differences for fatigue/recovery variables is shown in Figure 8. The preliminary meta-analysis with the inclusion of outliers (extremely high heterogeneity: diamond ratio = 5.11) is shown in the Appendix A Figure A2.

**Figure 9:**
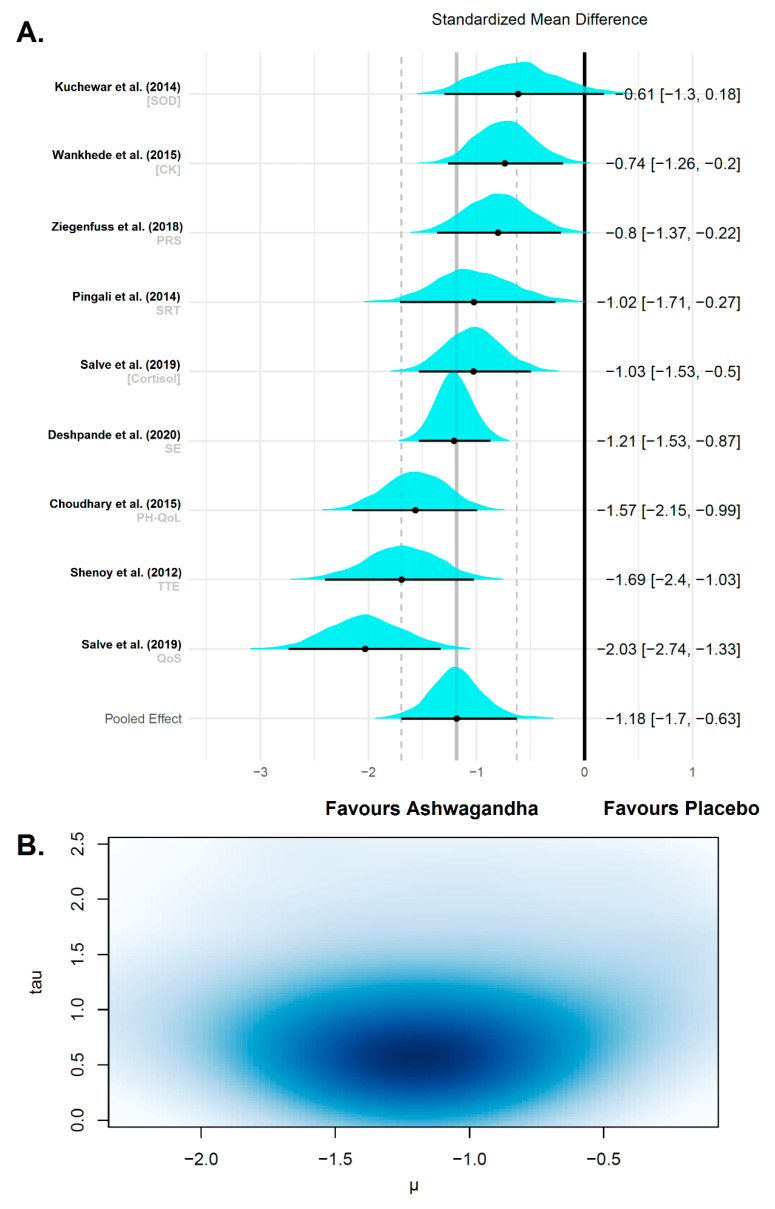
Forest plot of a random effect meta-analysis on fatigue/recovery variables after Ashwagandha supplementation. (**A**) The plot shows the names of the studies with their respective variable or multiple comparison/multiple outcomes on the left. On the right are the *θ_k_* and 95% CrI. The value of the overall weighted mean of effect sizes is drawn as a vertical line in the middle of the plot, which represents the reference line to test the null hypothesis in each study. The posterior distributions of the estimated effect sizes for each study are shown as cyan densities. The black circle represents the posterior mean, and the horizontal line extending from the point is the 95% CrI. The bottom row is the meta-analytic effect size (µ). (**B**) Multivariate kernel density estimation plot of the posterior distribution of μ (x-axis) and τ (y-axis) with the darker zone indicating increased plausibility of values.

Finally, after the exclusion of outliers, the pooled effect size in our Bayesian model was −1.18 (95% CrI from −1.70 to −0.63). Thus, there was a large overall effect size that favored the Ashwagandha interventions in this subgroup meta-analysis on fatigue/recovery-related variables. Our Bayesian model converged to a target distribution (Ȓ = 1). A medium between-study standard deviation was identified (τ = 0.66 (95% CrI −0.25–1.31)) with an absolute value of the true variance of τ^2^ = 0.43 (medium heterogeneity). The ECDF showed that the probability of the pooled effect being greater than 0.20 in a future observation is very high (99.9%), which suggests the effects of the Ashwagandha supplementation are very probable to be meaningful to reduce fatigue and to improve recovery. The results of this Bayesian random-effects meta-analysis is shown in Figure 9.

## 4. Discussion

### 4.1. Summary of Evidence

The aim of this systematic review and meta-analysis was to evaluate the effect of Ashwagandha supplementation on physical performance. A comprehensive systematic review was performed to report the different experimental designs and findings of the available body of evidence. Furthermore, using estimation statistics analysis and developing Bayesian hierarchical models, the meta-analysis of the included studies on three components of physical performance (strength/power, cardiorespiratory fitness and fatigue/recovery) revealed that comparing Ashwagandha administration and placebo resulted in a pooled effect size estimate substantially different from zero that favored the treatment effect. Collectively, the findings of this study demonstrated that Ashwagandha supplementation was more efficacious than placebo for improving physical performance. In addition, the implementation of a Bayesian approach, the assessment of the models’ integrity and the medium-to-low heterogeneity among the studies (both diamond ratio and τ^2^) provide certain confidence in the results that have been obtained. In fact, according to Kruschke and Liddell (2017), “Bayesian methods are well suited for meta-analytic modeling because meta-analytic models are a kind of hierarchical model and Bayesian methods are exceptionally useful for hierarchical models” [42].

Based on our results, we must highlight the following facts: (i) all input Markov chains had similar behavior for the studied variables (Ȓ = 1), which is sufficiently indicative of no problems in the model fit; (ii) although often hard to interpret, the estimation of the between-study standard deviation and variance (τ and τ^2^, respectively) offered certain advantages to the meta-analysis if we consider they are insensitive to the number of studies and the precision; and (iii) we were able to make reasonable inferences of probability on future observations based on the present evidence in the meta-analysis. With regard to Ashwagandha supplementation, we can be quite confident that future interventions may be at least in some way beneficial on the analyzed components of physical performance since the 95% credible intervals for the meta-analytic effect size lay completely on the “favoring” side of the intervention. In spite of the above, due to the low number of studies in each subgroup and the inclusion of multiple effect sizes from single studies (i.e., multiple outcomes), the pooled treatment effects should be interpreted with caution, and further research is needed to confirm the ergogenic effects of Ashwagandha.

It has been reported that Ashwagandha supplementation (between 240 and 600 mg daily) might enhance strength/power-related variables in healthy untrained individuals that are not frequently involved in exercise training and physical conditioning programs [19,31,38,40]. Only one study [20] has demonstrated that exercise adaptations to resistance training can increase after twelve weeks of Ashwagandha supplementation (500 mg o.d. qAM, Sensoril^®^ ≥10% withanolides & ≤0.5% withaferin-A) in strength-trained individuals. Similarly, the study of Raut et al. (2012), which was excluded from the meta-analysis since it was not placebo-controlled, concluded that Ashwagandha is not only safe at increasing doses of 750 to 1250 mg·day^−1^ but also has positive effects on muscle strength in physically active healthy individuals [32]. The changes reported in the analyzed studies include variables such as muscle strength, muscle size, serum testosterone, maximum velocity, absolute and relative power. Although more research in exercise-trained individuals is needed, this Bayesian meta-analysis showed that the probability of having a clinically significant increase in strength/power compared to placebo after the Ashwagandha supplementation is very high.

In regards to cardiorespiratory fitness, Pérez-Gómez and colleagues (2020) reported in a recent meta-analysis that Ashwagandha supplementation significantly increases VO_2max_ in both untrained and exercise-trained participants [18]. However, unlike their methodology, we did not exclude data from the meta-analysis because we based our analysis on changes from baseline, which might be more efficient and powerful than the comparison of post-intervention values solely according to the current version of the Cochrane Handbook for Systematic Reviews of Interventions [24]. Furthermore, the authors of this recent meta-analysis conducted a wrong extraction process of the data for their analysis. For example, Shenoy et al. [33] reported that three male subjects dropped out due to inconsistent attendance in training sessions (one from the placebo and two from the Ashwagandha group with a final sample size of *n* = 19 and *n* = 18, respectively); notwithstanding, Pérez-Gómez et al. performed the meta-analysis with the initial number of participants in each group (*n* = 20 each one). Moreover, Choudhary et al. [37] reported the following values of VO_2max_: 41.74 (5.21), 46.65 (6.29) and 47.41 (6.63) at baseline, week 8 and week 12, respectively; however, Pérez-Gómez et al. performed the meta-analysis with 46.65 (6.29) as value for the week 12. These flaws can be easily identified in Figure 3 and Table 2 of their published article. The results of our subgroup meta-analysis (six studies, seven effect sizes) showed a large, pooled treatment effect on cardiorespiratory fitness. Additionally, VO_2max_ and [Hb] increased significantly and were statistically different from placebo across the studies after 8–24 weeks of daily supplementation with 330–1000 mg of Ashwagandha. There is a high probability of having clinical significance in future observations, and it seems that the higher the dosage, the better the outcomes and more suitable for athletes and trained individuals, but further investigations are necessary.

Finally, the consumption of 120–1000 mg Ashwagandha per day has been shown to reduce fatigue and optimize recovery in healthy individuals. The selected studies that were included in this systematic review showed improvements in physical health (a component of the quality-of-life test), muscle fatigue (increasing both times to exhaustion and perceived recovery), muscle damage/soreness (less CK levels and increase in SOD), sleep recovery (better overall quality of sleep) and stress levels (considerable reductions in cortisol levels). Very high heterogeneity was obtained among the studies (diamond ratio = 5.11) after the first exploratory meta-analysis. This was due to the inclusion of two outliers: (i) an extremely positive impact on the physical health component of the quality-of-life test after Ashwagandha supplementation, as reported by Deshpande et al. [41] (very high effect size); and (ii) a very large reduction on cortisol concentrations in the Ashwagandha group compared to placebo (−23.3% vs. +0.5%, respectively) observed by Lopestri et al. [40]. Albeit these reported changes were in agreement with other references [37,39], their inclusion in the meta-analysis resulted in severe heterogeneity; however, we should bear in mind that this high between-study variance is itself informative as may be seen as opportunities to explore ways to improve the current status of literature. After the removal of these outliers, the Bayesian meta-analysis model revealed that, in comparison to the placebo, the pooled effect of Ashwagandha supplementation was very large (−1.18 (95% CrI from −1.70 to −0.63)) and that future observations are very probable to be meaningful on the reduction of fatigue and the improvement of recovery.

### 4.2. Potential Mechanisms of Action

Currently, there are no studies describing the molecular mechanisms of Ashwagandha extracts associated with improving physical performance and reducing muscle fatigue in humans. However, the positive effects of the aqueous root extract of Ashwagandha on the muscle strength/power, cardiorespiratory fitness and fatigue/recovery that were described by most of the studies included in this systematic review might be due, in part, to the antioxidant properties of this plant. It is well known that adequate physiological levels of reactive oxygen species (ROS) are required for the processes of physiological adaptation to exercise training; notwithstanding, overtraining, low energy availability or inadequate sleep hygiene might increase the ROS production, which in turn can negatively affect the time course of exercise-induced adaptations [43,44,45]. For instance, these molecules have been shown to induce structural modifications of myofibrillar proteins, which affect their function (e.g., low sensibility to intracellular Ca^2+^). In this sense, several studies have demonstrated that antioxidant supplementation (such as N-acetylcysteine) may delay muscle fatigue during prolonged exercise in humans [46,47,48], but high doses have also been associated with reductions in exercise benefits. Thus, a common rationale for the increase in strength and power has been the possible optimization in the process of muscle adaptation and recovery after a physical effort, which has a practical implication on the frequency, and therefore the volume, of the training.

Even though animal and in vitro studies cannot be directly extrapolated to human effects and their results have to be interpreted with attention [49], several animal and cell studies have identified potential molecular targets of the secondary metabolites of this herbal extract that could be involved in the regulation of oxidative stress at the cellular level. Palliyaguru et al. (2016) revealed that withaferin A is a potent inducer of Nrf2, a transcription factor that regulates the expression of antioxidant enzymes in response to oxidative stress, through the activation of the PTEN/PI3K/Akt pathway, in a murine model with induced liver toxicity [50]. Likewise, Yan et al. (2018) demonstrated that treatment with withaferin A of myoblast cells exposed to simulated ischemia/reperfusion or treated with H_2_O_2_ increased cell survival by inducing the expression of proteins with antioxidant activity HO-1, SOD2, SOD3 and Prdx-1, dependent on the activation of Akt [51]. The cytoprotective effect of Ashwagandha through the activation of Nrf2 has been corroborated in different cellular contexts and in different combinations of herbal extracts [52,53,54]. On the other hand, several anabolic and catabolic signaling pathways regulating muscle protein synthesis and energy metabolism might be affected after the administration of Ashwagandha. Of these, the NF-κB signaling pathway has been involved in the regulation of myogenesis in skeletal muscle in humans and in animal models. Although several authors have described a promyogenic function after the activation of this pathway, others have demonstrated the potent inhibitory effect of myogenesis and muscle regeneration, suggesting a complex regulation of this signaling pathway during muscle formation [55]. Several studies have shown the effect of withanolides on the regulation of NF-κB transcriptional activity in different in vivo and in vitro experimental models [10,56,57]. Ichikawa et al. (2006) reported that withaferin A and viscosalactone, along with their acetylated derivatives, block this signaling pathway by inhibiting the phosphorylation and degradation of IB, blocking the expression of NF-κB-related genes [56]. Kaileh et al. (2017) demonstrated that withaferin A directly inhibits IKK kinase activity, which phosphorylates IκBα prior to its ubiquitination and degradation, through a redox mechanism sensitive to thiol alkylation in a MEK1/ERK1 dependent manner [10]. Likewise, withaferin A and withanolide D blocked the angiogenic activity of endothelial cells by inhibiting the degradation of IB and inducing the expression of the antioxidant enzyme HO-1 [57]. Thus, although there is no evidence describing the mechanisms associated with increased muscle strength/power after supplementation with Ashwagandha root extract, withaferin A, among other secondary metabolites, have been identified as regulators of different cellular contexts that may favor myogenesis and oxidative metabolism in humans.

In terms of the cardiorespiratory fitness and endurance performance, it has been reported that Ashwagandha supplementation contributes to the increase of [Hb] and hematological markers (mean corpuscular hemoglobin concentration [MCH], mean cell hemoglobin concentration [MCHC], and mean corpuscular volume [MCV]) while preventing oxidative stress [34,35]. These physiological effects might be crucial mechanisms to explain the increase in VO_2max_. Schumacher et al. [58] reported higher MCV and MCH in the competition phase compared to the off-season period in the elite cyclists. Borges et al. [59] also showed a substantial increase in MCV and MCH in elite kayakers after a period of high-intensity training. While Rietjens et al. (2002) reported a low correlation between [Hb] and VO_2max_ obtained in cycle ergometer and treadmill in triathletes [60], some research has shown a correlation between these variables in athletes trained at altitude [61] and between the [Hb] and the ability to exercise [62,63]. Additionally, some authors highlight the importance of monitoring hematological status and possible iron supplementation since, throughout the year, several athletes show values of [Hb], hematocrit and ferritin below the normal range [60]. Interestingly, Shenoy et al. (2012) reported a higher increase in time to exhaustion in men in comparison to women (10.7% vs. 4.3%, respectively), which resulted in higher outcomes of VO_2max_ for men (16.1% vs. 9.0%, respectively). This implies that men responded more to supplementation than women, perhaps mediated by an Ashwagandha effect on the endocrine system [33]. It should also be noted that: (i) athletes and physically active women are more susceptible to iron deficiency, which can contribute directly and indirectly to energy deficiency and changes in performance due to changes at the hematological level [64]; and (ii) supplementation with Ashwagandha extracts (600 mg per day delivering 21 mg withanolide glycoside) could not only counteract the production of ROS during exercise but also increase the levels of testosterone in healthy males [65] (probably through metabolic pathways of steroid hormone biosynthesis involving the conversion of saponins or sterane derivatives found in Ashwagandha [6] to testosterone and derivatives such as DHEA), which would justify the differences in physical performance between men and women if we consider the well-documented erythrogenic effects of testosterone [66].

Finally, considering the importance of rest and sleep in the processes of adaptation to physical effort, some of the reviewed references support the fact that supplementation with Ashwagandha might optimize the quality of sleep. In fact, Ashwagandha root extract (300 mg twice daily for 10 weeks) has been found to have the potential to induce sleep and improve sleep quality in patients with insomnia without presenting side effects [67]. This could not only favor the processes of adaptation to exercise but also accelerate rehabilitation and physical readaptation after a musculoskeletal injury. In this sense, a randomized, double-blind, placebo-controlled clinical trial showed that supplementation with 125 mg and 250 mg of Ashwagandha, both taken bis in die in patients with knee joint pain over a 12 week period, had a significant reduction in treatment efficacy and tolerability outcomes compared to baseline and placebo (with better results when taking 250 mg twice per day) while any significant gastrointestinal distress was detected [68].

**Figure 10:**
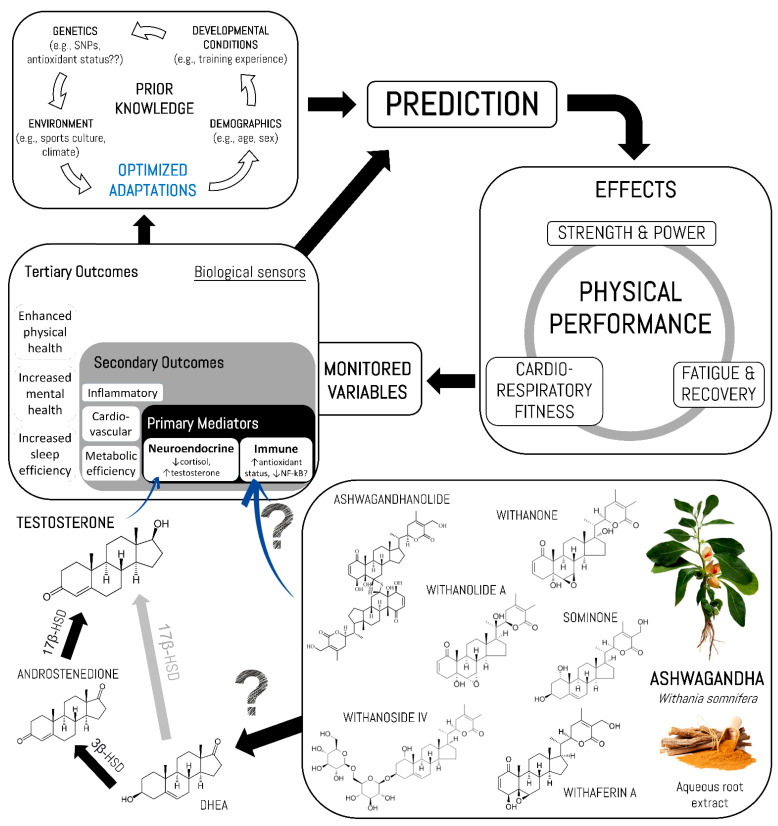
Potential mechanisms of action with feedback control of the Ashwagandha effects on physical performance variables. 3β-HSD: 3β-hydroxysteroid dehydrogenase; 17β-HSD: 17β-hydroxysteroid dehydrogenase; DHEA: dehydroepiandrosterone; SNP: single nucleotide polymorphism.

Based on a model of predictive regulation [69], Ashwagandha might help overcome the allostatic overload and accelerate exercise-induced adaptations by the activation of actuator variables that modify the allostatic state (e.g., allostatic response to physical exercise) [45], albeit further standardized research is needed to have a better comprehension of its effects under certain bio-psycho-environmental situations. Figure 10 represents the potential mechanisms of action with feedback control of the Ashwagandha effects on physical performance variables.

### 4.3. Practical Applications

Our comprehensive systematic review and Bayesian meta-analysis showed that Ashwagandha supplementation protocols between 120 mg and 1250 mg per day might enhance physical performance. In regards to timing, seven studies asked participants to supplement with Ashwagandha once a day as follows: four studies every morning, two studies every afternoon or evening, and one study implemented every night at bedtime supplementation. On the other hand, six studies have implemented doses *bis in die* (i.e., twice per day—early in the morning and at night).

Interestingly, higher doses were preferred for trained individuals and athletes; therefore, it seems that 300–500 mg twice per day (morning and before sleep) might be a safe and effective supplementation protocol for both female and male undergoing strenuous resistance or endurance training, but we are aware that further research is needed to individualize accordingly. Lower doses (≤300 mg once a day) might be considered for non-physically active individuals, those with no training experience and/or those engaging in exercise programs.

The concentrations of withanolides were between 5% and 35% (only one study reported 42 mg by HPLC quantification) and less than 0.5% of withaferin A. The most frequent commercial products used in the studies were KSM-66^®^, Shoden^®^ and Sensoril^®^. We would like to highlight the common phytochemical variability that can be found in all herbal extracts, including Ashwagandha. In fact, Sangwan et al. [70] concluded that frequent oscillations might be due to factors such as (i) heterogeneous bioresources (wild and/or harvested); (ii) physiological and ecological variations in plantations; (iii) harvest and postharvest collection operations; (iv) biomass processing and product manufacturing process; and (v) unregulated, contaminated and undescribed supplements. Recently, new analytical methods (e.g., high-performance thin-layer chromatography, HPTLC) have been reported to rapidly and selectively detect and quantify certain withanolides and phenolic compounds present in different components of Ashwagandha (root, stem and leaf) [71], which will ensure the quality and reliability of the products used in future studies.

Although we did not perform a rigorous analysis of discontinuation rates and patient satisfaction, it should be noted that none of the clinical trials have found serious adverse effects from the consumption of Ashwagandha in the doses and length administered. Actually, Raut et al. (2012) demonstrated that the aqueous root extract of Ashwagandha seems to be safe on hematological and biochemical organ function tests. More recently, Salve et al. (2019) noted that 600 mg of Ashwagandha per day was well tolerated with no adverse events reported by the participants during eight weeks.

### 4.4. Future Directions

According to the results obtained in the present systematic review, and considering recent results that suggest that the Ashwagandha root extract can be used to control body mass in adults under chronic stress [72], future studies may evaluate the effect of Ashwagandha supplementation during nutritional and exercise intervention programs to improve body composition for body esthetics, health or sports performance.

Studies are also needed at different altitudes and environmental conditions (e.g., heat and humidity) since it may be a strategy to reduce environmental impact and maximize adaptations to the exercise. A registered clinical trial will allow us to know more about the effects on endurance performance (NCT03596307), but further research is still needed in other sports.

More research comparing sex differences is needed, considering that it seems the production of testosterone derivatives (e.g., DHEA) from withanolides may lead to important differences. In this sense, the positive benefits on the hormonal profile (DHEA-S and testosterone) in overweight men over 50 s [65], on the immediate and general memory, executive function, attention and speed of information processing in subjects aged 50 with mild cognitive impairment [73], and on the quality of life, mental alertness and quality of sleep in healthy men and women over 70 s [74], makes the Ashwagandha root extract a nutritional alternative to optimize physical condition, bone and muscle health while preventing loss of muscle mass and strength in elderly.

Further research is required to evaluate and identify the most important secondary metabolites of Ashwagandha in order to generate enriched supplements while optimizing and standardizing the methods of quantitative analysis in broad-spectrum products (e.g., KSM-66), which will increase the reproducibility of studies in terms of dose and concentration of withanolides.

Finally, this is an open field for new studies on different exercise- and sports-related variables, but considering our experience dealing with missing data, low-quality biased studies or data misinterpretation, researchers are encouraged: (i) to register their protocols in any of the current organizations or collaboration networks (e.g., US Clinical Trials, EU Clinical Trials Register, OSF International, Campbell Collaboration, among others); and (ii) to follow robust reporting guidelines to increase quality and transparency of clinical research (visit https://www.equator-network.org/ accessed on 28 February 2021). Far from being a meaningless value judgment, this is a call to action to report in detail relevant information within the current paradigm of open science.

### 4.5. Limitations

This systematic review with meta-analysis has certain limitations: (i) the low number of studies published to date; (ii) we limited the databases for data extraction to PubMed/Medline, ScienceDirect, and Google Scholar; (iii) there were considerable differences in the exercise training experience among the participants included in the analyzed studies; (iv) although τ^2^ is insensitive to the number and the precision of the included studies, further analytical approaches are necessary with other types of heterogeneity measures when more placebo-controlled randomized-clinical trials are available. In spite of the above, the Bayesian method has tremendous advantages compared to the frequentist method if we consider that the estimates allow us to discuss probabilities, which makes it more intuitive, direct, coherent, accurate, and flexible than frequentist approaches [42].

## 5. Conclusions

The findings of this comprehensive systematic review and Bayesian meta-analysis revealed that Ashwagandha supplementation was more efficacious than placebo for improving variables related to strength/power, cardiorespiratory fitness and fatigue/recovery in healthy men and female. In fact, the probability of at least a small effect size on physical performance favoring subjects supplemented with Ashwagandha is very high (>95%). However, more comparable studies in exercisers/athletes are needed to derive a more stable estimate of the true underlying effect of the consumption of this aqueous herbal extract in trained individuals.

## Figures and Tables

**Table 1:** Evidence of the effects of Ashwagandha supplementation on physical performance.

Reference	Sample (F:M)	Age/ BMI	Ashwagandha (WS) Supplementation				Analyzed Variables	Change	Conclusions
			Groups	Design/ Length	Dosage (mg)	(Withanolides)			
Sandhu et al., 2010 [31]	40 (18:22)	20.6 (2.5) 21.9 (2.2)	WS (*n* = 10) TA (*n* = 10) WS+TA (*n* = 10) Placebo (flour, *n* = 10)	RCT-SB/ 8 weeks	500 (o.d. qAM)	ARE (ND)	V_Max_ (m·s^−1^) ^a^ W_absolute_ (W) ^b^ W_relative_ (W·kg^−1^) ^b^ VO_2máx_^c^	+2.9% †* +8.8% †* +10.1% †* +6.8% †*	WS may be useful against physical exhaustion and to improve speed, lower limb muscle strength and neuromuscular coordination.
Raut et al. 2012 [32]	18 (6:12)	24.3 (2.1) 24.2 (2.7)	WS (*n* = 18)	CT-O/ 30 days	750 per day × 10 days, 1000 per day × 10 days and 1250 per day × 10 days (b.i.d.)	ARE 8:1 (ND)	Muscle strength (kg): Handgrip Quadriceps Back extensors RPE-Borg (VAS)	+8.0% ^NS^ +21.5% † +15.4% † −26.9% ^NS^	WS supplementation is not only safe but has positive effects on muscle strength.
Shenoy et al., 2012 [33]	40 (20:17)	19.6 (1.4) 19.6 (1.9)	WS (*n* = 18) Placebo (starch, *n* = 19)	RCT/ 8 weeks	1000 (500 b.i.d.)	ARE (ND)	VO_2máx_^c^ TTE (min) RER ^c^	+13% †* +7.2% †* +1.8% ^NS^	First study to report significant improvements in the sports performance of elite athletes.
Malik et al., 2013 [34]	32 (0:32)	17.4 (1.7) 20.9 (2.9)	WS (*n* = 16) Placebo (sugar, *n* = 16)	RCT-SB/ 8 weeks	500 (q.h.s.)	ARE (ND)	VO_2máx_^d^ [Hb]	+6.67% †* +5.14% †*	WS supplementation for eight weeks improves VO_2max_ and [Hb] in hockey players.
Kuchewar et al., 2014 [35]	30 (20:10)	18 to 45 years old	WS (*n* = 11) *Guduchi* (*n* = 10) Placebo (CaCO_3_, *n* = 9)	RCT-DB/ 6 meses	1000 (500 b.i.d.)	ARE (ND)	[Hb] MCV (µm^3^) MCHC (%) MCH (pg) MDA SOD	+6.29% † +8.12% † +4.45% † +8.15% † −18.2% † +18.1% †	WS contributes to the increase of [Hb] and hematological markers while preventing oxidative stress.
Pingali et al., 2014 [36]	20 (0:20)	24.9 (4.18) 22.38 (1.1)	WS (*n* = 10) Placebo (carbohydrates mixture, *n* = 10)	RCT-DB Crossver/14 days (14 days wash-out)	1000 (500 b.i.d.)	ARLE Sensoril^®^ ≥10% withanolides and ≤0.5% withaferin-A	Reaction time: SRT CDT DSST DVT	−6.2% †* −3.39% †* −8.14% †* −3.16% †*	Fourteen days of WS supplementation decreases reaction time compared to placebo, indicating a positive effect on cognitive and psychomotor function.
Choudhary et al., 2015 [37]	49 (ND)	20 to 45 years old/ 18.5 to 24.9 kg·m^−2^	WS (*n* = 25) Placebo (saccharose, *n* = 24)	RCT-DB/ 3 months	600 (300 b.i.d.)	ARE KSM‑66^®^ 5% withanolides	VO_2máx_^e^ Quality of life test: Physical health Psychological health	+6.2% †* +14.7% †* +19.6% †*	WS root extract increases cardiorespiratory endurance and improves quality of life in healthy athletic adults.
Wankhede et al., 2015 [19]	50 (0:50)	28.0 (8.0) /NA	WS (*n* = 25) Placebo (starch, *n* = 25)	RCT-DB/ 8 weeks	600 (300 b.i.d.)	ARE KSM‑66^®^ 5% withanolides	PB 1-RM KE 1-RM Arm (cm^2^) Chest (cm) Thigh (cm^2^) % BF ^f^ [T] ↑[CK] 24/48	+138% †* +51.9% †* +17.1% †* +3.32% †* +8.0% † +16.0% †* +15.2% †* −98.9% †*	WS supplementation during a resistance training program increases muscle strength and size while accelerating post-exercise recovery in untrained subjects.
Tripathi et al., 2016 [38]	30 (0:30)	27.3 (2.48) 22.9 (1.44)	WS330 (*n* = 10) WS500 (*n* = 10) Placebo (starch, *n* = 10)	RCT-O/ 28 days	330 and 500 per group (o.d. qAM)	ARE (ND)	Max Dist ^g^ V_av_^g^ (km·h^−1^) V_max_ (km·h^−1^) ^g^ Handgrip (kg): VO_2máx_^h^	WS330: +15.7% †* +15.9 †* +9.3% ^NS^ +4.95% † +5.40% † WS500 +15.9% †* +16.2% †* +12.0% † +4.93% † +6.09% †	WS supplementation for 28 days increases muscle strength and cardiorespiratory capacity in healthy men.
Ziegenfuss et al., 2018 [20]	38 (0:38)	24.4 (4.2) 26.2 (3.4)	WS (*n* = 19) Placebo (flour, *n* = 19)	RCT-DB/ 3 months	500 (o.d. qAM)	ARLE Sensoril^®^ ≥10% withanolides and ≤0.5% withaferin-A	SQ 1-RM SQ peak power (W) ^i^ SQ mean peak (W) ^i^ SQ Reps 65%-RM BP 1-RM BP peak power (W) ^i^ BP mean peak (W) ^i^ BP Reps 65%-RM Time-trial 7.5 km (s) ^j^ PRS (VAS) [Hb]	+18.0% †* +8.45% †* +4.64% † +22.8% † +13.6% † +11.3% †* +11.6% †* +28.1% † −21.2% † +14.4% † −1.93% †	WS supplementation (500 mg·day^−1^) for twelve weeks of a resistance training program improves upper and lower limb muscle strength and power and perceived recovery in recreationally active subjects.
Salve et al., 2019 [39]	58 (ND)	31.1 (7.5) /NA	WS250 (*n* = 19) WS600 (*n* = 20) Placebo (starch, *n* = 19)	RCT-DB/ 8 weeks	250 and 600 per group (o.d. qAM)	ARE KSM‑66^®^ 5% withanolides	PSS-10 [cortisol] HAM-A QoS	WS250: −33.7% †* −16.5 †* −13.0% NS −41.0% †* WS600 −38.3% †*⁑ −32.6% †*⁑ −16.3% †* −46.0% †*⁑	Eight weeks of supplementation of aqueous WS root extract was associated with a significant reduction of stress levels and improved the overall quality of life.
Lopestri et al., 2019 [40]	60 (23:37)	42.2 (2.44) 24.6 (0.60)	WS (*n* = 30) Placebo (toasted rice, *n* = 30)	RCT-DB/ 60 days	240 (o.d. qPM)	ARLE Shoden^®^ ≥35% withanolide glycosides	HAM-A DASS-21 [Cortisol] [DHEA-S] [T]	−40.8% †* −30.0 † −23.3% †* −8.15% †* +11.4 †	WS reduces anxiety levels, [cortisol] and [DHEA-S], with a non-significant tendency to increase [testosterone] in men.
Deshpande et al., 2020 [41]	150 (78:72)	36.8 (10.9) 25.0 (3.9)	WS (*n* = 75) Placebo (rice powder, *n* = 75)	RCT-DB/ 6 weeks	120 (o.d. qPM)	ARLE Shoden^®^ ≥35% withanolide glycosides (42 mg HPLC)	RSQ-W SOL (min) ^k^ WASO ^k^ TTS (min) ^k^ SE (%) ^k^ Quality of life test: Physical health Psychological health	+72% †* −27.2% †* −14.7% †* +4.8% †* +4.6% †* +13.1 † +11.8 †	120 mg of WS extract increases the overall quality of sleep (time and efficiency) and increases the quality of life at the physical and psychological levels.

Data are expressed as mean (SD). The change values are expressed as the percentage change of the group supplemented with Ashwagandha according to the formula: ((postpre)/pre) × 100. ↑[CK] 24/48: increase in serum creatine kinase concentration (U/L) from 24 h to 48 h post-exercise; [cortisol]: serum cortisol concentration (µg·dL^−1^); [DHEA-S]: serum DHEA sulfate concentration (µg·dL^−1^); [Hb]: blood hemoglobin concentration; [T]: serum testosterone concentration (ng·dL^−1^);% BF: body fat percentage; 1-RM: one-repetition maximum (kg); ARE: aqueous root extract; ARLE: aqueous root and leaves extract; b.i.d.: bis in die—twice a day; BMI: body mass index (kg·m^−2^); BP: bench press; CDT: choice discrimination test; CT-O: open-label clinical trial; DASS-21: depression anxiety stress scales-21; DSST: digit symbol substitution test; DVT: digit vigilance test; F: female; HAM-A: Hamilton anxiety rating scale; KE: knee extension; M: male; MCH: mean corpuscular hemoglobin; MCHC: mean cell hemoglobin concentration; MCV: mean corpuscular volume; MDA: malondialdehyde (nmol·mL^−1^); Max Dist: maximum distance (km); NA: not available; o.d.: omne in die—once a day; PRS: perceived recovery scale; PSS-10: perceived stress scale; qAM: quaque ante meridiem—every morning; q.h.s.: quaque hora somni—every night at bedtime; QoS: quality of sleep; qPM: quaque post meridiem—every afternoon or evening; RCT-O: open-label randomized clinical trial; RCT-SB: single-blinded randomized clinical trial; RCT-DB: double-blinded randomized clinical trial; Reps 65%-RM: as many repetitions as possible with a load of 65% RM; RER: respiratory exchange ratio; RPE-Borg: rating of perceived exertion; RSQ-W: restorative sleep questionnaire-weekly version (0–100); SE: sleep efficiency; SOD: superoxide dismutase (U·g^−1^ Hb); SQ: squat; SOL: sleep onset latency; SRT: simple reaction time; TA: *Terminalia arjuna*; TTE: time to exhaustion; TTS: total time of sleep; VAS: visual analog scale; V_Max_: maximum velocity; V_av_: average velocity; W_absolute_: absolute power; W_relative_: relative power; WASO: wake after sleep onset (min); WS: *Withania somnifera* (Ashwagandha). ^a^ Photoelectric sensor; ^b^ contact mat; ^c^ ergospirometry (mL·kg^−1^·min^−1^); ^d^ Cooper’s 12 min run test (mL·kg^−1^·min^−1^); ^e^ Léger’s test (L·min^−1^); ^f^ tetrapolar bioelectrical impedance analysis; ^g^ six-minute cycle ergometer exercise test; ^h^ YMCA cycle ergometer submaximal test (mL·kg^−1^·min^−1^); ^i^ linear position transducer system TENDO; ^j^ cycle ergometer; ^k^ ActiSleep^®^; † significant difference in comparison to baseline; * significant difference in comparison to placebo; ⁑ significant difference in comparison to the other Ashwagandha group; ^NS^ no significant change.

## Data Availability

The data supporting this systematic review and meta-analysis are from previously reported studies and datasets, which have been cited.

## Appendix A

The exploratory subgroup meta-analysis of the seven initial studies (including the outlier study) on the evaluation of cardiorespiratory fitness after Ashwagandha supplementation is shown in Figure A1.

The exploratory subgroup meta-analysis of the initial studies (including two outliers) on the evaluation of fatigue/recovery after Ashwagandha supplementation is shown in Figure A2.
